# Molecular Docking and Dynamics Simulations Reveal the Antidiabetic Potential of a Novel Fucoxanthin Derivative from *Chnoospora minima*

**DOI:** 10.3390/md23120471

**Published:** 2025-12-09

**Authors:** Sachini Sigera, Kavindu D. Theekshana, Sathmi G. Dinanja, Pasindu Eranga, Nayanatharie Karunathilake, Shamali Abeywardhana, Laksiri Weerasinghe, Tharindu Senapathi, Dinithi C. Peiris

**Affiliations:** 1 Genetics & Molecular Biology Unit, Faculty of Applied Sciences, University of Sri Jayewardenepura, Nugegoda 10250, Sri Lanka; tharushika.sigera@gmail.com (S.S.);; 2 Department of Zoology, Faculty of Applied Sciences, University of Sri Jayewardenepura, Nugegoda 10250, Sri Lanka; dinuharakt@gmail.com (K.D.T.); pasinduerangaonline@gmail.com (P.E.);; 3 Department of Chemistry, Faculty of Applied Sciences, University of Sri Jayewardenepura, Nugegoda 10250, Sri Lanka

**Keywords:** fucoxanthin derivative, *Chnoospora minima*, antidiabetic activity, α-amylase inhibitor, α-glucosidase inhibitor, molecular docking, molecular dynamics

## Abstract

Type 2 diabetes mellitus (T2DM) is a chronic metabolic disorder requiring safer and more effective therapeutic alternatives. This study investigates a novel fucoxanthin derivative isolated from the marine brown alga *Chnoospora minima* using a comprehensive in silico approach. Molecular docking revealed that the derivative exhibited higher binding affinities toward α-amylase (–9.4 kcal/mol) and α-glucosidase (–8.0 kcal/mol) compared to the reference drug acarbose (–8.5 and –7.4 kcal/mol, respectively). Pharmacokinetic analysis predicted good intestinal absorption and P-gp inhibition (0.894) and moderate plasma clearance (7.864 mL/min/kg), while toxicity predictions classified it in toxicity class 3, with no respiratory or ocular toxicity. Drug-likeness evaluation showed only one Lipinski and one Veber rule violation, common for natural products. Molecular dynamics simulations conducted for 100 ns using NAMD 3.0 confirmed stable protein–ligand complexes with average RMSD values of ~1.3 Å and ~1.8 Å for α-amylase and α-glucosidase, respectively, and consistent hydrogen bonding profiles. Structural analysis identified a substitution of the allene bond with an unsaturated ketone at the C8′ position as a key contributor to enhanced enzyme interaction. The findings suggest that this fucoxanthin derivative is a promising natural candidate for T2DM therapy and warrants further investigation through lab experiments (in vitro and in vivo).

## 1. Introduction

Type 2 diabetes mellitus (T2DM) is one of the most common metabolic disorders and a significantly growing global health threat characterized by chronic hyperglycemia. The leading cause of T2DM is attributed to insulin resistance or impaired insulin secretion, resulting in neuropathy, nephropathy, retinopathy, and an increased risk of cardiovascular diseases [1]. These complications significantly reduce quality of life and impose a substantial burden on healthcare systems [2]. Diabetes ranks as the third leading cause of death worldwide and is linked to serious complications. Type 2 diabetes, which accounts for over 90% of diabetes cases, is characterized by hyperglycemia resulting from insulin deficiency and resistance [3]. The global surge in type 2 diabetes and obesity is attributed to rapid urbanization and unhealthy dietary patterns, with more than 90% of diabetes patients categorized as overweight or obese [4].

Recent data indicate that 23% of adults in Sri Lanka have diabetes, while an additional 30.5% are in a pre-diabetic state, reflecting the higher prevalence in South Asia and imposing a significant economic burden [5,6].

Postprandial hyperglycemia plays a vital role in the advancement of type 2 diabetes and complications. Inhibition of carbohydrate digestion occurs by blocking the activity of enzymes such as α-amylase and α-glucosidase, which are essential for carbohydrate hydrolysis and to control postprandial blood glucose levels [7]. Though the current medicines enhance insulin secretion, suppress hepatic glucose production, and inhibit renal glucose reabsorption, these drugs are associated with minor to severe side effects, including gastrointestinal disturbances, weight gain, and hypoglycemia, or limited efficacy [8]. In this context, natural plant-based alternative approaches to T2DM management are considered for their multi-target activity, better biocompatibility, and fewer side effects.

Marine algae have been recognized as a rich source of bioactive compounds with therapeutic potential [2,9]. Carotenoids, specifically fucoxanthin, found predominantly in brown algae, have shown remarkable antidiabetic potential by inhibiting key digestive enzymes, α-amylase and α-glucosidase [8,9]. Recent publications have highlighted their therapeutic benefits, attracting researchers [10]. The authors also emphasized that brown algae are rich in bioactive compounds, including peptides, polyphenols, phytosterols, and polysaccharides such as alginate, fucoidan, and laminarin.

Previously, we showed that the Sri Lankan marine brown alga *Chnoospora minima,* belonging to the family Scytosiphonaceae, exhibits significant antidiabetic activity by inhibiting α-amylase and α-glucosidase enzymes [10]. Additionally, in our efforts to isolate the bioactive compound associated with diabetes, we successfully isolated a new fucoxanthin derivative from the methanol extract of *C. minima* [11]. Several fucoxanthin analogues are reported in marine organisms, including fucoxanthinol, amarouciaxanthin A, and halocynthiaxanthin, which typically arise through metabolic deacetylation, oxidation, or rearrangement of the parent fucoxanthin molecule. Fucoxanthin analogues retain the characteristic allenic bond, 5,6-epoxide, and conjugated backbone of fucoxanthin [12,13]. In contrast, our isolated derivative exhibits distinct structural modifications not previously described, underscoring the novelty of our compound.

Nevertheless, the antidiabetic efficacy of the isolated compound has yet to be fully verified. Therefore, our primary objective in this study is to evaluate the potential of the newly isolated fucoxanthin derivatives as antidiabetic agents through an extensive in silico approach. We utilized molecular docking, toxicity prediction, ADMET profiling, and molecular dynamics (MD) simulations to explore its interactions with crucial diabetes target enzymes, α-amylase and α-glucosidase. Additionally, we aimed to assess its potential as a promising candidate for antidiabetic drug development.

## 2. Results

### 2.1. Phytochemicals

#### 2.1.1. Total Phenolic Content (TPC)

The total phenolic content (TPC) of the crude methanol extract and its fractions of *C. minima* ranged from 2.96 ± 0.41 to 58.11 ± 4.28 mg GAE/g of extract. The ethyl acetate fraction (58.11 ± 4.28 mg GAE/g) indicated the highest phenolic content, followed closely by the crude methanol extract (57.01 ± 6.12 mg GAE/g). The chloroform fraction (36.42 mg GAE/g) exhibited a moderate level, while the aqueous fraction (19.90 ± 2.11 mg GAE/g) and the hexane fraction (2.96 ± 0.41 mg GAE/g) contained comparatively lower phenolic contents. Thus, the phenolic content of the crude methanol extract and its fractions decreased in the order: ethyl acetate fraction > crude methanol extract > chloroform fraction > aqueous fraction > hexane fraction (Table 1).

#### 2.1.2. Total Flavonoid Content (TFC)

The total flavonoid content (TFC) of the crude methanol extract and fractions of *C. minima* varied between 0.21 ± 0.06 and 5.24 ± 1.01 mg QE/g of extract. The ethyl acetate fraction recorded the highest flavonoid content (5.24 ± 1.01 mg QE/g), followed by the chloroform fraction (3.31 ± 0.04 mg QE/g) and the aqueous fraction (1.05 ± 0.07 mg QE/g). The crude methanol extract (0.79 ± 0.04 mg QE/g) and the hexane fraction (0.21 ± 0.06 mg QE/g) showed comparatively lower flavonoid contents. Accordingly, the flavonoid content increased in the order: hexane fraction < crude methanol extract < aqueous fraction < chloroform fraction < ethyl acetate fraction (Table 1).

### 2.2. Antidiabetic Activities

#### 2.2.1. α-Amylase Activity

**Figure 1:**
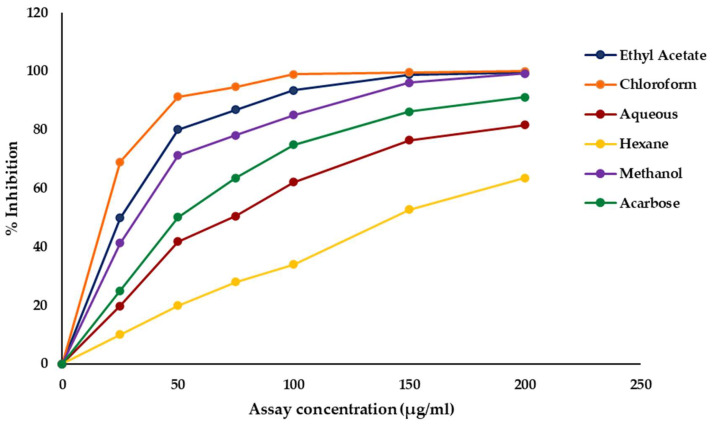
Dose–response curves of α-amylase inhibitory activities of crude methanol extract and fractions of *Chnoospora minima*.

The α-amylase inhibitory activities of the crude methanol extract and its solvent fractions of *C. minima* were evaluated, and the results are presented in Figure 1. All the tested extracts showed a concentration-dependent increase in inhibitory activity against α-amylase.

Among the fractions, the chloroform fraction exhibited the most potent inhibitory effect with the lowest IC_50_ value of 5.34 ± 0.32 µg/mL, indicating potent α-amylase inhibition, even surpassing the standard drug acarbose (IC_50_ = 72.41 ± 0.24 µg/mL). The ethyl acetate fraction also showed potent activity with an IC_50_ of 30.56 ± 0.56 µg/mL, followed by the methanol fraction (45.63 ± 0.04 µg/mL). The aqueous fraction demonstrated moderate activity (IC_50_ = 92.12 ± 1.20 µg/mL), whereas the hexane fraction displayed the weakest inhibitory effect with the highest IC_50_ value of 149.31 ± 0.94 µg/mL (Table 2).

#### 2.2.2. α-Glucosidase Activity

**Figure 2:**
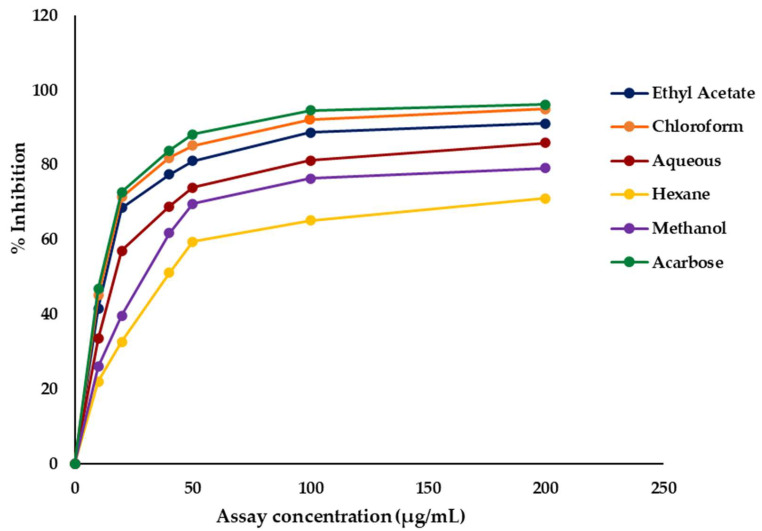
Dose–response curves of α-glucosidase inhibitory activities of crude methanol extract and fractions of *Chnoospora minima*.

The α-glucosidase inhibitory activities of the crude methanol extract and its solvent fractions of *C. minima* were investigated, and the results are illustrated in Figure 2. All extracts exhibited a concentration-dependent increase in inhibitory activity. Among the fractions, the chloroform fraction displayed the most potent inhibition with an IC_50_ value of 6.02 ± 0.18 µg/mL, followed by the ethyl acetate fraction (14.78 ± 0.26 µg/mL). The aqueous fraction also showed considerable activity (IC_50_ = 36.92 ± 1.06 µg/mL), whereas the methanol fraction exhibited moderate inhibition (IC_50_ = 58.88 ± 2.01 µg/mL). The hexane fraction demonstrated the weakest activity with an IC_50_ of 83.92 ± 0.54 µg/mL (Table 2).

When compared with the standard drug acarbose (IC_50_ = 1.02 ± 0.07 µg/mL), the chloroform fraction (6.02 ± 0.18 µg/mL) and the ethyl acetate fraction (14.78 ± 0.26 µg/mL) exhibited relatively strong inhibitory potential, whereas the aqueous, methanol, and hexane fractions were markedly less active (Table 2).

### 2.3. Fractionation, and Compound Isolation

Since the chloroform fraction exhibited strong antidiabetic potential, inhibiting α-amylase (IC_50_ = 5.34 ± 0.32 µg/mL) and α-glucosidase (IC_50_ = 6.02 ± 0.18 µg/mL), it was further fractionated by silica gel 60 chromatography (F_A_–F_F_). Fraction F_D_ exhibited high potency of α-amylase (IC_50_ = 93.43 ± 0.45 µg/mL) and α-glucosidase (IC_50_ = 47.25 ± 0.46 µg/mL) inhibitory activity, and it was subjected to further purification using Sephadex LH20 chromatography (F_D1_–F_D8_). Subfraction F_D3_ exhibited the most potent enzyme-inhibitory activity and was subsequently purified by reverse-phase HPLC, yielding a single major compound [11].

**Figure 3:**
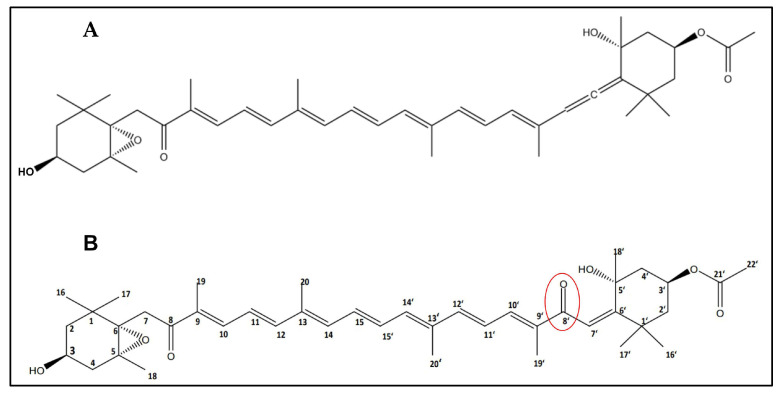
Structures of fucoxanthin compounds (**A**): Original fucoxanthin compound and (**B**): the newly isolated fucoxanthin derivative (The red circle highlights the modified functional group (carbonyl group) introduced in the derivative compared to the original fucoxanthin structure).

Structural elucidation of the purified compound was performed using ^1^H and ^13^C NMR spectroscopy, supported by 2D experiments (COSY, HSQC, and HMBC) [11]. Based on the spectral information, the molecular formula of the compound was identified as “C_42_H_58_O_7_”. The NMR structural elucidation data confirmed that the isolated compound was a “fucoxanthin derivative” (Table 3). The newly isolated fucoxanthin derivative possesses an additional unsaturated ketone at C8′, replacing the allene bond (C=C=C) of the structure (Figure 3B). Substitution of the C8′ allenoic bond made the isolated compound structurally unique.

### 2.4. Pharmacokinetic Profiles and Drug-likeness Characteristics of the Optimized Ligand

**Figure 4:**
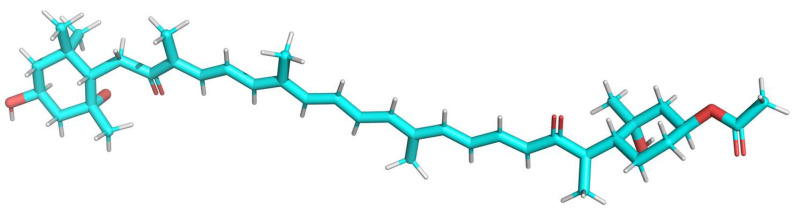
The 3D structure of the optimized Fucoxanthin Derivative (FD) is depicted, with carbon atoms colored blue, oxygen atoms red, and hydrogen atoms white.

The pharmacokinetic profile and drug-likeness analysis of the optimized ligand (isolated fucoxanthin derivative) (Figure 4) results are represented in Table 4. The ligand demonstrates good human intestinal absorption, poor Caco-2 cell permeability, and potent P-glycoprotein inhibition, supporting a moderate absorption potential. Further, logS is 5.261, indicating poor aqueous solubility, which is typical for carotenoids. Regarding distribution, it exhibits moderate plasma protein binding and low blood–brain barrier permeability, indicating moderate distribution behaviours. Metabolic profiling predicts excellent inhibition and substrate affinity toward CYP1A2 and CYP2D6 enzymes, poor inhibition but excellent substrate activity for CYP2C19 and CYP2C9, and medium inhibitory potential against CYP3A4, collectively suggesting favorable metabolic properties. Excretion parameters indicate moderate plasma clearance and a short elimination half-life, reflecting rapid excretion. Toxicological predictions classify the compound under toxicity class 3, with moderate potential for AMES mutagenicity and positive skin sensitization, but no predicted respiratory toxicity or eye irritation, indicating an overall moderate toxicity risk.

### 2.5. Protein Structure Validation

**Figure 5:**
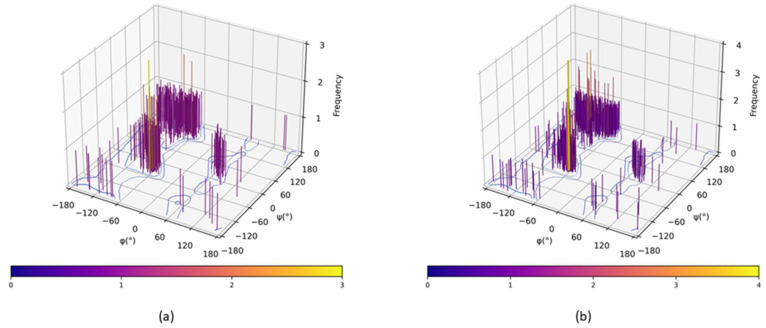
Standard 2D Ramachandran plots of the (**a**) optimized α-amylase and (**b**) optimized α-glucosidase models generated by RAMPAGE. The 3D bars represent the frequency of backbone torsion angles (φ and ψ) adopted by the residues.

The stereochemical quality of the energy-minimized α-amylase and α-glucosidase models was evaluated using the Ramachandran plot generated by RAMPAGE. (Figure 5; Table 5). The α-amylase model exhibited 97.61% (449 residues) of its residues in the favoured regions, 2.39% (11 residues) in the allowed areas, and 0% in disallowed regions. Similarly, the α-glucosidase model showed 96.02% (771 residues) in the favored areas, 3.74% (30 residues) in allowed regions, and a minimal 0.25% (2 residues) in disallowed regions.

The high percentage of residues in favoured regions (>95%) and the very low number in the disallowed areas indicate good stereochemical quality and acceptable backbone geometry for both models [14]. The 3D Ramachandran plots (Figure 5) further illustrate that the residues cluster within typical secondary structure regions, including α-helices and β-sheets, with few outliers. Overall, the Ramachandran analysis confirms that the optimized 3D models are stereochemically stable and suitable for subsequent analyses.

### 2.6. Molecular Docking

**Figure 6:**
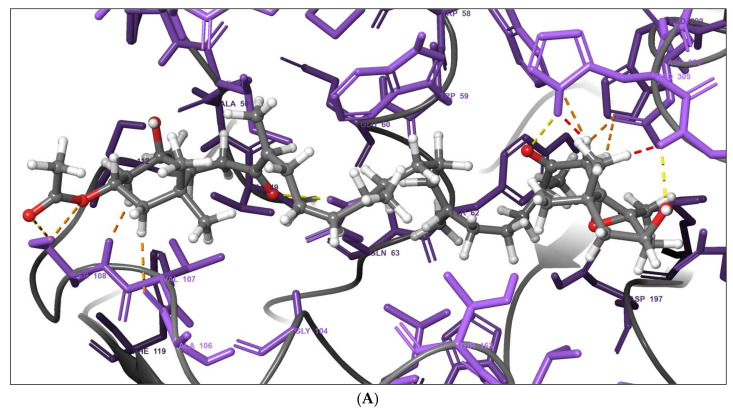

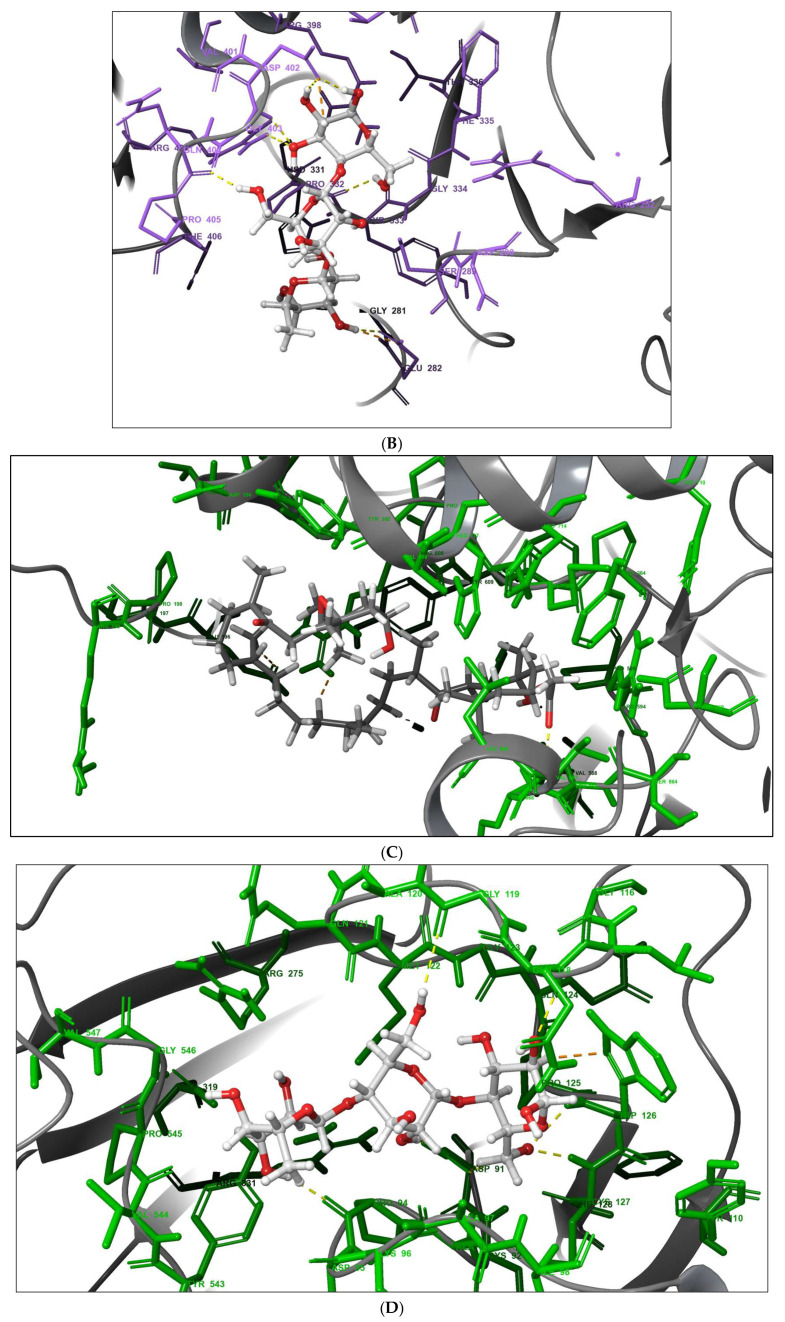
(**A**), 3D interaction diagram of α-Amylase–fucoxanthin derivative complex (**B**), 3D interaction diagram of α-Amylase–acarbose complex (**C**), 3D interaction diagram of α-Glucosidase–fucoxanthin derivative complex (**D**), 3D interaction diagram of α-Glucosidase–acarbose complex.

The molecular docking results of the fucoxanthin derivative and the reference drug, acarbose, against α-amylase and α-glucosidase are summarized in Table 6. The 3D binding interactions of the docked complexes are illustrated in Figure 6. The fucoxanthin derivative exhibited a higher binding affinity toward α-amylase (–9.4 kcal/mol) compared to Acarbose (–8.5 kcal/mol) (Table 6). It formed three hydrogen bonds with SER108, HSD305, and GLY306 at bond distances of 2.80 Å, 3.19 Å, and 3.28 Å, respectively (Figure 6A), and established 59 non-bonded contacts. In contrast, acarbose formed five hydrogen bonds with GLU282, ASP402 (two bonds), GLY403, and ARG421, along with 55 non-bonded contacts and a salt bridge interaction (Figure 6B). Similarly, the fucoxanthin derivative showed a higher binding affinity toward α-glucosidase (–8.0 kcal/mol) than acarbose (–7.4 kcal/mol) (Table 6). It formed a single hydrogen bond with VAL718 (2.84 Å), 67 non-bonded contacts, and a salt bridge interaction (Figure 6C). Acarbose formed five hydrogen bonds with ASP91, ALA93, PRO94, and GLN118 (two bonds), along with 61 non-bonded contacts (Figure 6D).

### 2.7. Molecular Dynamics (MD) Simulation

**Figure 7:**
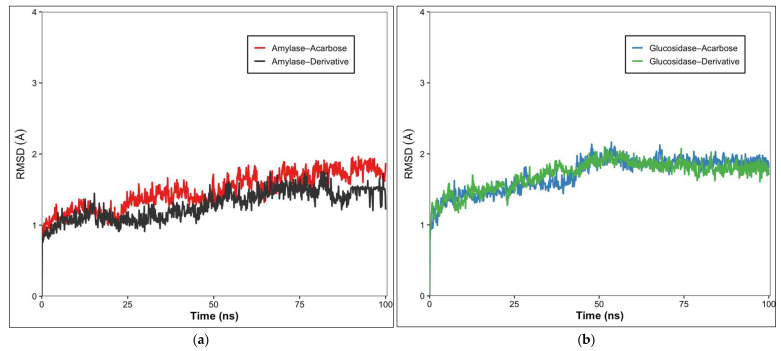
Root Mean Square Deviation (RMSD) plots of the molecular dynamics simulations for the docked complexes. (**a**) The α–amylase–fucoxanthin derivative complex is shown in black, the α–amylase–acarbose complex in red, and (**b**) the α–glucosidase–fucoxanthin derivative complex in blue, and the α–glucosidase–acarbose complex in green.

Root-mean-square deviation (RMSD) plots were generated to evaluate the structural stability and binding dynamics of the complexes following molecular dynamics (MD) simulations (Figure 7). During the initial phase of the production (0–20 ns), both the α–amylase–fucoxanthin derivative complex and the α–amylase–acarbose complex exhibited an initial increase in RMSD values, after which the trajectories began to stabilize. After 30 ns, the α-amylase-fucoxanthin derivative complex exhibited lower, more stable fluctuations, maintaining an average RMSD of approximately 1.3 Å throughout the simulation, indicating high structural stability. In contrast, the α–α-amylase–acarbose complex showed slightly higher RMSD values after 50 ns, averaging around 1.6 Å with more pronounced fluctuations, suggesting comparatively lower structural stability (Figure 7a).

When comparing the structural stability of the α-glucosidase-fucoxanthin derivative complex and the α-glucosidase-acarbose complex during the MD simulation, both complexes exhibited an initial increase in RMSD values during the first 10 ns (equilibration phase), after which the trajectories stabilized (Figure 7b). Throughout the remainder of the simulation, RMSD fluctuations were maintained around ~1.8 Å for the fucoxanthin derivative complex and ~2.0 Å for the acarbose complex. The α-glucosidase-fucoxanthin derivative complex exhibited a slightly more consistent, smoother RMSD trajectory, indicating greater structural stability. In contrast, the α-glucosidase-acarbose complex exhibited minor but noticeable fluctuations, particularly between 30 and 30–50 ns, suggesting transient conformational shifts during that period.

**Figure 8:**
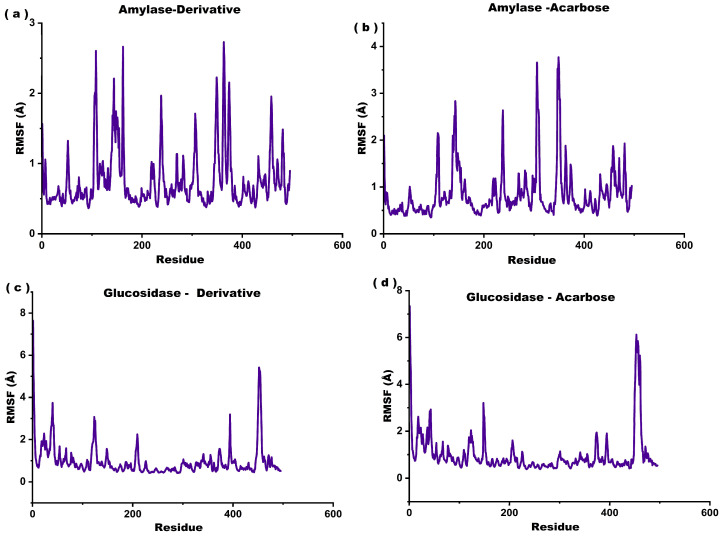
Root mean-square fluctuation (RMSF) plots of the molecular dynamics simulations for the docked complexes. (**a**) The α–amylase–fucoxanthin derivative complex, (**b**) the α–amylase–acarbose complex, (**c**) the α–glucosidase–fucoxanthin derivative complex, and (**d**) the α–glucosidase–acarbose complex.

**Figure 9:**
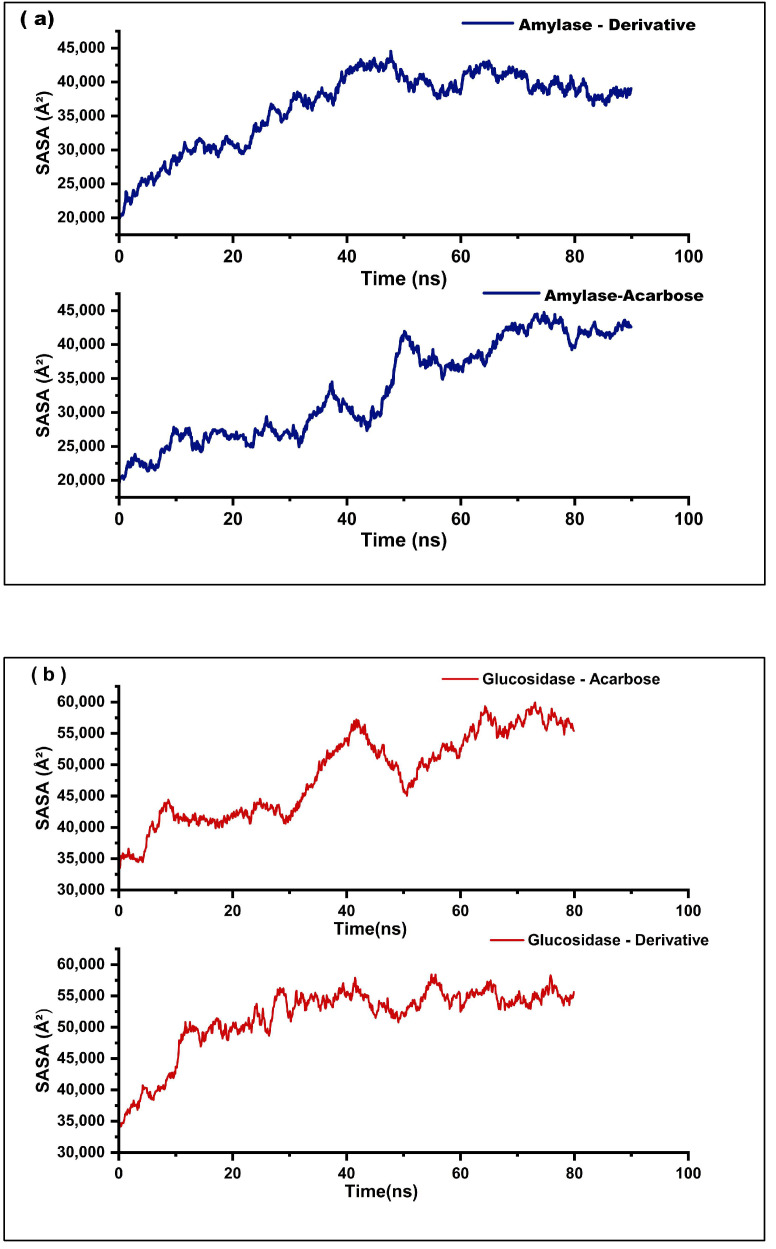
Solvent Accessible Surface Area (SASA) plots of the molecular dynamics simulations for the docked complexes. (**a**) The α-amylase-fucoxanthin derivative complex and α-amylase-acarbose complex (**b**) The α-glucosidase-fucoxanthin derivative complex and α-glucosidase-acarbose complex.

Root-mean-square fluctuation (RMSF) plots were generated to evaluate residue-level flexibility for each complex throughout molecular dynamics (MD) simulations (Figure 8). The RMSF values for both the α-amylase–fucoxanthin derivative complex and the α-amylase–acarbose complex range mainly between 0.5 and 2.5 Å, indicating moderate residue fluctuations (Figure 9a,b). α-amylase–acarbose complex shows higher peaks around residues ~150, ~300, and ~380 compared to α-amylase–fucoxanthin derivative complex, with values reaching up to ~3.8 Å, suggesting increased local flexibility in these regions (Figure 8b). In contrast, the α-amylase-fucoxanthin derivative complex exhibits relatively reduced fluctuations, with most residues staying below 2.5 Å, implying better structural rigidity and potentially greater conformational stability (Figure 8a).

The RMSF values for both the α-glucosidase–fucoxanthin derivative complex and the α-glucosidase–acarbose complex exhibit distinct patterns of residue flexibility across the protein structure (Figure 8c,d). The α-glucosidase–acarbose complex shows slightly sharper peaks near residues 30 and 400, extending to ~6.5 Å, indicating increased local flexibility in these regions (Figure 8d). In contrast, the α-glucosidase-fucoxanthin derivative shows broader but less intense fluctuations, with maximum values peaking around 6 Å (Figure 8c). The results suggest that the α-glucosidase-fucoxanthin derivative complex maintains a more stabilized backbone conformation compared to the α-glucosidase-acarbose complex.

To evaluate conformational stability and solvent exposure of the four complexes, SASA profiles were analyzed over the simulation time course (Figure 9a,b). In the α-amylase–acarbose complex, the SASA increased steadily from ~22,000 Å^2^ at the beginning to a peak of ~43,000 Å^2^ by 40 ns, followed by moderate fluctuations and stabilization around 37,000–38,000 Å^2^ toward the end of the 100 ns simulation. In contrast, the α–amylase–fucoxanthin derivative complex exhibited a similar starting SASA (~21,000 Å^2^), but with a sharper rise between 40 and 50 ns, reaching ~42,000 Å^2^ and maintaining a higher, relatively stable SASA throughout the remaining simulation (Figure 9a).

For the α-glucosidase-acarbose, SASA increased from ~34,000 Å^2^ to a peak of ~58,000 Å^2^ around 50 ns, followed by slight decreases and fluctuations, ultimately stabilizing near 55,000–56,000 Å^2^ by the end of the 85-ns run. The α-glucosidase-fucoxanthin derivative complex also showed a gradual increase in SASA from ~34,000 Å^2^ to ~57,000 Å^2^ by 40 ns, after which the values fluctuated slightly and stabilized between 52,000 and 54,000 Å^2^ (Figure 9b).

**Figure 10:**
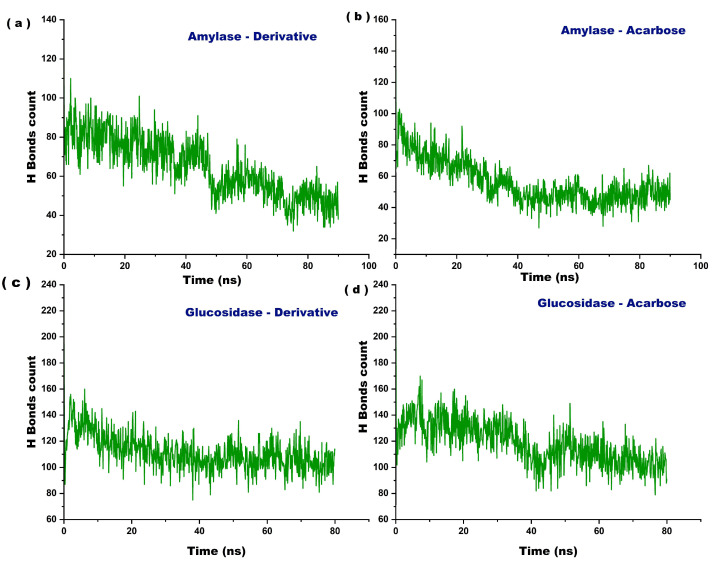
The hydrogen bond profile of the molecular dynamics simulations for the docked complexes. (**a**) α-amylase–fucoxanthin derivative complex, (**b**) α-amylase–acarbose complex, (**c**) α-glucosidase–fucoxanthin derivative complex, (**d**) α-glucosidase–acarbose complex.

To assess the stability and interaction strength within the protein–ligand complexes, hydrogen bond analysis was conducted throughout the molecular dynamics simulations (Figure 10). The α–amylase–fucoxanthin derivative showed a continuous decline in H-bond count, decreasing from approximately 90–100 at the start to around 40–50 by the end of the 100 ns simulation (Figure 10a). In contrast, the α-amylase–acarbose complex initially exhibited higher H-bonds (~100–120), which declined sharply during the first 40 ns but then stabilized around 50–60 ns (Figure 10b). α-Glucosidase–fucoxanthin derivatives exhibit the most consistent H-bonding behavior, maintaining many interactions (~130–160 initially, stabilizing around 110–120), reflecting strong and sustained structural integrity (Figure 10c). The α-glucosidase–acarbose complex also began with a high H-bond count (~140–160), but experienced a gradual decline with greater fluctuations, stabilizing around 100–110 toward the end of the simulation (Figure 10d).

## 3. Discussion

Type 2 diabetes mellitus (T2DM) continues to pose significant challenges to global health systems, with its prevalence steadily increasing worldwide [3]. The management of postprandial hyperglycemia remains a critical therapeutic target in controlling T2DM progression and preventing associated complications [8]. Polyphenols and flavonoids are important secondary metabolites known for their potent antioxidant and antidiabetic activities, primarily due to their ability to modulate carbohydrate-digesting enzymes and reduce oxidative stress. In the present study, in vitro data revealed that *Chnoospora minima* possesses a high phytochemical content, with the ethyl acetate (58.11 ± 4.28 mg GAE/g) and chloroform (36.42 ± 2.74 mg QE/g) fractions exhibiting the highest TPC) and TFC (ethyl acetate fraction 5.24 ± 1.01 mg GAE/g and chloroform fraction 3.31 ± 0.04 mg QE/g) values (Table 1). These fractions also demonstrated the strongest α-amylase (ethyl acetate fraction 30.56 ± 0.56 µg/mL and chloroform fraction 5.34 ± 0.32 µg/mL) and α-glucosidase (ethyl acetate fraction 14.78 ± 0.26 µg/mL and chloroform fraction 6.02 ± 0.18 µg/mL) inhibitory activities, indicating a clear relationship between phytochemical abundance and antidiabetic potential. Such correlations are well supported, as phenolic and flavonoid compounds contribute to enzyme inhibition, free-radical scavenging, and improved glucose metabolism [15,16]. Although the chloroform fraction exhibited the strongest α-amylase and α-glucosidase inhibitory activity, among solvent sub-fractions, potency was reduced. The superior potency exhibited by the original chloroform fraction is likely due to the synergistic effect of multiple bioactive constituents, including phenolics, flavonoids, and carotenoids present in the chloroform fraction [17]. Since our main objective was to isolate the single antidiabetic compound responsible for this activity, the chloroform fraction was selected for further purification.

Upon fractionation by silica gel and Sephadex LH-20 chromatography, the IC_50_ values of the subfractions (F_A_-F_D8_) decreased because the synergistic effects were lost during separation. Subfraction F_D3_ was identified as the most active individual compound-containing fraction, highlighting that it represents the major contributor to the antidiabetic activity of the chloroform fraction. The purification of subfraction F_D3_ yielded a fucoxanthin derivative with the molecular formula C_42_H_58_O_7_. Fucoxanthin and its derivatives are major carotenoids in brown algae and are known for diverse biological activities, including antioxidant, anti-obesity, and antidiabetic effects [18].

The primary structural difference between the fucoxanthin derivative and native fucoxanthin lies in the substitution of a characteristic allene bond with an unsaturated ketone group at the C8′ position (Figure 3). While native fucoxanthin contains an allenoic bond (C=C=C) adjacent to the polyene chain, the derivative replaces this feature with a conjugated ketone, a modification not previously reported among marine carotenoids. Known fucoxanthin analogues such as fucoxanthinol (results from deacetylation), amarouciaxanthin A (involves deacetylation and oxidation of the terminal ring), and halocynthiaxanthin (retains an intact allene group) undergo structural changes in other regions of the molecule but consistently preserve the allene functionality [13]. The introduction of the C8′ ketone is therefore structurally distinct and may influence molecular rigidity, electron distribution, and conformational stability. Hence, the introduction of the C8′ ketone enhances the molecule’s ability to form more stable interactions with protein targets without disrupting the core carotenoid structure. This unique structural modification, combined with the absence of comparable transformations in established fucoxanthin metabolic pathways, supports the novelty and potential functional relevance of the derivative [12,19].

Based on these findings, the isolated fucoxanthin derivative (C_42_H_58_O_7_) was further validated for its antidiabetic potential using a series of in silico approaches, including pharmacokinetic profiling, molecular docking, and molecular dynamics (MD) simulations. The MD simulations were performed under standard in vitro physiological conditions (neutral pH and physiological ionic strength) to evaluate the intrinsic stability of the fucoxanthin derivative–enzyme complexes. Collectively, our study provides compelling evidence that the isolated fucoxanthin derivative may act as an effective inhibitor of key carbohydrate-hydrolyzing enzymes to manage postprandial hyperglycemia.

The pharmacokinetic profile predicted by ADMETlab 3.0 for the derivative indicated positive human intestinal absorption (HIA: Yes). The predicted Caco-2 cell permeability (log Papp = −4.947) suggests moderate to low passive diffusion across the intestinal epithelium. The overall optimistic HIA prediction, coupled with significant P-gp inhibition (P-gp inhibitor = 0.894), suggests potentially acceptable oral absorption characteristics [20]. The discrepancy between the positive HIA prediction and the low Caco-2 permeability may result from active transport processes or limitations of the prediction models, underscoring the need for experimental in vitro and in vivo studies to confirm oral bioavailability. Further experimental validation would be necessary to confirm the precise oral bioavailability [20]. Distribution parameters (PPB = 78.24%, BBB = 0) and metabolism predictions (excellent CYP1A2/CYP2D6 interactions, medium CYP3A4 inhibition) align with desirable drug-like features, albeit with one Lipinski and one Veber rules violation due to molecular weight (>500 Da) and TPSA (>140 Å^2^) [21]. Such violations are common for natural products and can be mitigated through formulation strategies or prodrug approaches [22]. Toxicity profiling classified the compound as class 3, with moderate Ame’s mutagenicity (0.708), positive skin sensitization, and no respiratory or ocular toxicity [23], indicating an acceptable safety margin for preclinical development. Nevertheless, the positive Ames test and class 3 toxicity suggest that careful structural optimization or additional safety studies may be required before clinical translation. Overall, the derivative’s ADMET profile is comparable to that of other marine-derived carotenoids under investigation [23], which often exhibit similar molecular complexity and pharmacokinetic challenges. However, with suitable structural or formulation modifications, this derivative demonstrates potential as a viable antidiabetic drug candidate (Table 4).

Structural validation of the protein models via Ramachandran plot analysis confirmed their high stereochemical quality, with 97.61% and 96.02% of residues in favoured regions for α-amylase and α-glucosidase, respectively (Figure 5, Table 5). These values exceed the 90% threshold recommended for reliable molecular docking studies [14], indicating that the models are well-refined and suitable for docking analyses.

The stronger binding affinity of the fucoxanthin derivative toward α-amylase (–9.4 kcal/mol) and α-glucosidase (–8.0 kcal/mol) compared to acarbose (–8.5 and –7.4 kcal/mol, respectively) can be attributed to the substitution of the characteristic allene bond with a conjugated ketone at the C8′ position (Table 6). This structural modification introduces a polar C–O bond due to the significant electronegativity difference between carbon and oxygen, generating a dipole with δ+ on carbon and δ− on oxygen. Such polarity enhances ligand-enzyme interactions by enabling more favorable hydrogen bonding, ion-dipole interactions, and dipole-induced dipole interactions, particularly with residues in the enzyme’s active sites [24].

In α-amylase, the derivative forms three hydrogen bonds with SER108, HSD305, and GLY306 at bond distances of 2.80–3.28 Å, while establishing 59 non-bonded contacts, exceeding those of acarbose (55). For α-glucosidase, a hydrogen bond with VAL867 (2.84 Å) and 67 non-bonded contacts suggest that the dipole and overall structural conformation enable the derivative to engage effectively with the residues. This bonding is typically considered non-polar (Table 6). Their interactions are likely to arise from the dipole-enhanced stabilization of the ligand within the active site, thereby improving binding affinity despite fewer hydrogen bonds than in acarbose.

Overall, the C8′ ketone not only increases molecular rigidity but also optimizes electrostatic and van der Waals interactions with key residues, providing a mechanistic explanation for the superior inhibitory potential of the fucoxanthin derivative against carbohydrate-hydrolyzing enzymes.

When benchmarked against known phytochemicals, the derivative’s docking scores compare favourably: Withaferin-A exhibited –9.79 kcal/mol against α-amylase in a recent study [25], and native fucoxanthin showed –7.0 kcal/mol in docking against α-amylase [26,27], and quercetin 3-rhamnoside demonstrated –8.6 kcal/mol for α-amylase [27]. Thus, the derivative’s –9.4 kcal/mol for α-amylase places it among the more potent natural inhibitors, exceeding native fucoxanthin and matching Withaferin-A closely. In contrast, its –8.0 kcal/mol for α-glucosidase surpasses that of acarbose and aligns with the top flavonoid inhibitors. This comparative analysis highlights that the structural modification in our derivative, specifically the replacement of the allenoic bond with an unsaturated ketone at C8′, likely contributes to its enhanced binding affinity.

Interestingly, despite forming fewer hydrogen bonds than acarbose, the fucoxanthin derivative established a greater number of non-bonded contacts (59 with α-amylase and 67 with α-glucosidase) (Table 6), suggesting that hydrophobic interactions, van der Waals forces, and π-interactions are the primary contributors to complex stabilization. The results are consistent with previous findings where carotenoid compounds, such as β-carotene, relied predominantly on non-bonded interactions for stable binding to proteins [28]. Additionally, the presence of a salt bridge in the α-glucosidase complex further enhances the binding through electrostatic interactions (Figure 6).

The root mean square deviation (RMSD) analyses demonstrated that both the α-amylase–fucoxanthin derivative and α-glucosidase–fucoxanthin derivative complexes achieved stability after the initial equilibration phase, with lower average RMSD values (1.3 Å and 1.8 Å, respectively) compared to the corresponding acarbose complexes (1.6 Å and 2.0 Å) (Figure 7). These lower RMSD values indicate more stable binding interactions, corroborating the superior binding affinities observed in the docking studies. Molecular dynamics studies have shown that RMSD values below 2 Å are generally indicative of stable protein–ligand complexes with minimal backbone deviations from the reference structure [29]. Notably, the α-amylase-fucoxanthin derivative complex exhibited particularly low RMSD fluctuations, suggesting a “lock-and-key” type of binding that maintains structural integrity over time. This binding stability is crucial for sustained enzyme inhibition and therapeutic efficacy.

Root Mean Square Fluctuation (RMSF) analyses from MD simulations indicate that derivative-bound enzyme complexes exhibit reduced atomic mobility compared to those bound with Acarbose. In α-amylase, most residue fluctuations in the derivative complex remained below 2.5 Å. In contrast, the acarbose-bound complex displayed pronounced peaks reaching approximately 3.8 Å near residues ~150, ~300, and ~380 (Figure 8b). Similarly, in α-glucosidase, the derivative complex exhibited broader but less intense fluctuations with a maximum around 6.0 Å, compared to sharper, more localized peaks up to 6.5 Å in the acarbose-bound form (Figure 8d). Reduced RMSF values suggest increased conformational rigidity, often associated with enhanced binding stability and prolonged residence time due to sustained ligand–protein interactions. These findings imply that the derivative may more effectively stabilize flexible loops and active-site regions, potentially lowering the ligand dissociation rate and improving inhibitory efficacy relative to acarbose [1].

SASA analysis showed generally stable solvent-exposed surface areas. The α-amylase-derivative complex (~42,000 Å^2^) maintained a higher SASA than its acarbose counterpart. In comparison, the α-glucosidase-derivative complex (57,000 Å^2^) exhibited a slightly more compact structure with a lower SASA than its acarbose complex, both of which are indicative of stable conformations. Consistent SASA profiles reflect a balance between ligand encapsulation within the binding pocket and appropriate solvent exposure, facilitating both strong binding and solvation shell stability [30]. The relatively stable SASA value obtained for the α-glucosidase–derivative complex suggests greater structural compactness and reduced fluctuations, indicating high solvent-exposure stability, which may contribute to its favorable binding and potential bioactivity profiles (Figure 9). Overall, these SASA profiles demonstrate how the complexes maintain structural compactness while balancing solvent exposure, a balance essential to their stability.

Among the other complexes, the α-glucosidase–derivative complex displayed the most consistent hydrogen bonding behaviour, maintaining a high number of interactions (~130–160 initially, stabilizing around 110–120), reflecting a strong and sustained structural integrity (Figure 10). This complex demonstrated the most stable hydrogen-bonding network, suggesting that it forms more stable, structurally consistent complexes than its counterparts [31].

The fucoxanthin derivative demonstrated stronger binding affinities for α-amylase and α-glucosidase than the standard drug acarbose, with stable interactions throughout molecular dynamics simulations. It also showed favourable pharmacokinetic properties, an acceptable safety profile, and promising drug-likeness, despite minor rule violations typical of natural compounds. These results suggest its strong potential as an effective antidiabetic agent.

In addition to its pharmacological potential, the marine origin of the fucoxanthin derivative offers a sustainable advantage. Brown algae species such as *Chnoospora minima* can be cultivated through environmentally responsible aquaculture methods that have been shown to minimize ecological impact while ensuring consistent biomass production. This aspect of sustainability is particularly relevant in the context of drug development for chronic diseases such as diabetes, where long-term therapeutic use necessitates reliable and eco-friendly sourcing of bioactive compounds [32].

In the future, the fucoxanthin derivative can be further investigated through comprehensive in vitro and in vivo studies to validate its antidiabetic efficacy and molecular mechanisms of action. These evaluations will be essential to confirm its potential as a therapeutic agent. Further research on structural modifications to enhance its pharmacokinetic properties, such as solubility, bioavailability, and metabolic stability, and to improve its overall drug-likeness is warranted. Additionally, exploring its activity against other diabetes-related molecular targets may broaden its therapeutic application. With continued optimization and validation, these derivatives represent a safe, effective, and natural alternative to conventional synthetic drugs for the management of type 2 diabetes mellitus.

## 4. Materials and Methods

### 4.1. Collection and Preparation of Algae Samples

*Chnoospora minima* samples were manually collected from Kalpitiya, Puttalam District, North-Western Province, Sri Lanka (6°40′54.19″ N: 80°80′51.78″ E). A voucher specimen was deposited in the Department of Zoology, Faculty of Applied Sciences, University of Sri Jayewardenepura, Sri Lanka.

The collected algal samples were thoroughly cleaned by sequential washing with fresh water, 10% HCl, and finally distilled water to remove sands, epiphytes, and other associated organic matter. The samples were then freeze-dried (lyophilized) and homogenized using a mechanical blender (Waring commercial blender, McConnellsburg, PA, USA). The resulting powdered material was stored at −20 °C until further extraction [33].

### 4.2. De-polysaccharide Crude Methanol Extraction and Solvent-Solvent Partition

The homogenized *C. minima* samples were extracted with 70% methanol sonication at 25 °C for three cycles of 90 min each. The extracts were centrifuged, and the resulting supernatants were filtered under vacuum through a celite bed packed in a Buchner funnel (diameter 70 mm, porosity 3). The solvent was subsequently removed under reduced pressure using a rotary evaporator (BUCHI, Rotavapor, R-300, New Castle, DE, USA).

The crude methanolic extract was redissolved in 70% ethanol (*v*/*w*, 1:25), shaken on an orbital shaker, and incubated overnight. The de-polysaccharide methanolic extract was concentrated under reduced pressure and freeze-dried to obtain a powdered sample stored at −20 °C. [34]

The de polysaccharide crude methanolic extract was suspended in deionized water and partitioned sequentially with solvents of increasing polarity: n-hexane, chloroform, and ethyl acetate in a separating funnel. This process yielded the hexane, chloroform, ethyl acetate, and aqueous fractions. Organic fractions were concentrated under reduced pressure, and the aqueous fraction was freeze-dried [34].

### 4.3. In Vitro Quantification of Phytochemicals

Total phenolic content was measured using the Folin–Ciocalteu reagent [35], reacting 20 µL of algae extracts and fractions (5–10 mg/mL) with diluted reagent, neutralized with sodium carbonate solution (10% *w*/*v*), and incubated at room temperature for 30 min. Absorbance was recorded at 765 nm, and phenolic content was quantified using a gallic acid standard curve [35,36].

The total flavonoid content was determined by the AlCl_3_ colorimetric method: algae extracts and fractions dissolved in methanol (5–10 mg/mL) were mixed with 100 µL of 2% AlCl_3,_ incubated at room temperature for 10 min, and the absorbance was recorded at 415 nm. The quercetin standard curve was used to determine total flavonoid content [36].

### 4.4. In Vitro Antidiabetic Activities

#### 4.4.1. α-Amylase Inhibitory Activity

Algal extracts and fractions were tested over a concentration range of 0.05–2 mg/mL. The extracts and the fractions of *C. minima* were diluted in 100 mM sodium acetate buffer (pH 6). Briefly, a reaction volume of 1 mL containing 200 μL of the sample, 40 μL of starch (1%, *w*/*v*), and 50 μL of the enzyme (5 μg/mL) in 100 mM sodium acetate buffer (pH 6.0) was incubated at 40 °C for 15 min in a shaking water bath. After the incubation period, 0.5 mL of DNS (3,5-dinitrosalicylic acid) reagent was added, and the mixture was heated in a boiling water bath for 5 min until the color developed. The reaction mixture was cooled in an ice-water bath, and the absorbance was measured at 540 nm using a 96-well microplate reader. A sample negative was carried out identically without adding the enzyme. Control experiments were conducted by replacing extracts with 200 µL of 100 mM sodium acetate buffer. Acarbose was used as the standard antidiabetic drug [37].

The capacity to inhibit the α-amylase enzyme by 50% (IC50) was calculated from the dose–response curves, and α-amylase inhibitory activity or % inhibition was calculated using the following equation.

Inhibition (%) = [AC − (ASP − ASN)/AC] × 100(1)

AC is the absorbance of the control, ASP is the absorbance of the sample, and ASN is the absorbance of the sample negative/sample blank.

#### 4.4.2. α-Glucosidase Inhibitory Activity

Algal extracts and their fractions were tested over a concentration range of 0.05–2 mg/mL. Ten µL of the sample (100 mM acetate buffer, pH 5.8), 20 µL of 100 mM acetate buffer, 25 mU/mL of the α-glucosidase enzyme, and 50 µL of PNPG (p-nitrophenyl-α-D-glucopyranoside) solution (6 mg/mL) were incubated at 37 °C for 35 min. Then, the reaction was stopped by adding 50 µL of 10% sodium carbonate solution, and the absorbance was recorded at 400 nm. The reaction mixture without extract was used as the control, and acarbose was used as the standard.

The capacity to inhibit the α-glucosidase enzyme by 50% (IC50) was calculated from the dose–response curves by linear regression, and α-glucosidase inhibitory activity % inhibition was calculated using the equation guveb ub sectuib 4.4.1. [38].

### 4.5. Bioassay-Guided Fractionation, Compound Isolation, and Structural Elucidation

The chloroform fraction of *Chnoospora minima* has been selected for bioassay-guided fractionation to isolate the bioactive compound, based on in vitro hypoglycemic activities and α-amylase and α-glucosidase inhibitory activities.

The chloroform fraction was subjected to column chromatography, and the resulting subfractions were combined according to thin-layer chromatography. The pooled fraction was further purified using Sephadex LH-20, and the active compound was finally isolated by C18 reverse-phase HPLC. Structural elucidation of the purified compound, identified as a fucoxanthin derivative, was achieved using proton (1H NMR) and carbon (13C NMR) [11].

### 4.6. Ligand Preparation and Structure Optimization

We created the 2D structure of the isolated fucoxanthin derivative, which, using ACD/ChemSketch (Freeware 2022.2.2) (Version C45E41, build 130928, 17 December 2024), was based on a reference structure derived from NMR [39]. The constructed molecule was saved in MOL format and subsequently converted to PDB format using PyMol 2.5.2 (https://pymol.org/, 12 November 2024) [40]. Optimization of the novel ligand was performed using the CHARMM-GUI Ligand Reader & Modeller tool (https://www.charmm-gui.org/?doc=input/ligandrm, 30 November 2024) with CHARMM General Force Field (CGenFF) parameters, yielding a minimized 3D structure [41].

Subsequently, the 3D structure of the reference drug, Acarbose, was retrieved from the PubChem database (https://pubchem.ncbi.nlm.nih.gov/, 15 November 2024) [42] in SDF format (CID: 41774), and optimization was performed using the CHARMM-GUI Ligand Reader & Modeller tool [41].

### 4.7. Pharmacokinetics and Drug-likeness Prediction

The open-source tool ADMETlab 3.0 (https://admetlab3.scbdd.com/, 2 January 2025) was utilized to evaluate the pharmacokinetic profiles of isolated fucoxanthin derivatives, covering absorption, distribution, metabolism, excretion (ADMET), and drug-likeness properties [43]. Subsequently, the ligand’s toxicity was predicted using the ProTox 3.0 online server (https://tox.charite.de/protox3/, 2 January 2025) [44].

### 4.8. Protein Structures Retrieval and Optimization

We retrieved the 3D structures of the target proteins, human pancreatic α-amylase (PDB ID: 1B2Y) and α-glucosidase (PDB ID: 5NN8), from the Protein Data Bank (PDB) server (https://www.rcsb.org/, 8 November 2024) [45]. The preparation of the protein structures by adding missing atoms, correcting protonation states, and optimizing the overall geometry using the PDB Reader and Manipulator tool from CHARMM-GUI [46]. The protonated protein structures were energy-minimized using the CHARMM36 force field, and the final structures were saved in PDB format [46].

### 4.9. Molecular Docking Study

We conducted protein-ligand docking using the PyRx Virtual Screening Tool (version 0.8) [47], which integrates AutoDock Vina 1.1.2 [48]. The optimized 3D structures of the ligands (fucoxanthin derivative and acarbose) and the proteins (human pancreatic α-amylase and α-glucosidase) were used for docking. The PDB files for both ligands and proteins were converted to the extended PDB format (PDBQT). Protein structure preparation included the removal of water molecules, the addition of polar hydrogens, and the assignment of Kollman charges. In contrast, ligand preparation included defining rotatable bonds and calculating Gasteiger charges. Docking was performed using a grid box size of 67.8 × 85.9 × 76.3 for α-amylase and 97.2 × 98.1 × 110.7 XYZ points for α-glucosidase, with a grid spacing of 1 Å. The grid centre was set at (X, Y, Z) with grid spacings of 18.725, 22.216, and 50.163 for α-amylase and −1.573, −17.898, and −21.039 for α-glucosidase.

We selected the best-docked conformations based on the lowest binding affinity, and each ligand-protein interaction was saved in PDB format for 3D visualization in PyMOL 2.5.2 [2]. The detailed analysis of the protein-ligand interactions in the best docked conformation was performed using PDBsum (http://www.ebi.ac.uk/thornton-srv/databases/pdbsum, 4 December 2024) [49]

### 4.10. Molecular Dynamics (MD) Simulation

MD simulation of the docked complexes was performed using the NAMD 3.0 [50] version with the CHARMM36m force field [51]. The docked complexes that exhibited the lowest binding affinity were prepared using the CHARMM-GUI Solution Builder [46] The complex structures were solvated in a rectangular box using the TIP3P water model and neutralized by adding K^+^ and Cl^−^ ions to replicate physiological conditions, with a pH of 7.0 [52,53]. Energy minimization was conducted using the steepest descent algorithm to eliminate steric clashes, followed by gradual heating to 303.15 K. Equilibration was then performed under the NPT ensemble, maintaining constant temperature and pressure using Langevin dynamics [54]. For the production MD simulation, the system was simulated for 100 ns under the NPT ensemble with a timestep of 2 fs, with a trajectory recorded every 10 ps. The analysis of the simulated trajectories included evaluating root-mean-square deviation (RMSD), root-mean-square fluctuation (RMSF), solvent-accessible surface area (SASA), and hydrogen bonds.

### 4.11. Data Analysis and Visualization

Statistical analyses were conducted using R software 4.1.3, Microsoft Excel 2016, and Minitab 17.1. Data visualization and graphical analyses were performed using visual molecular dynamics software (VMD 1.9.3), PyMol 2.5.2, and Origin 2022b for Windows.

## 5. Conclusions

This study highlights the promising antidiabetic potential of an isolated fucoxanthin derivative from *Chnoospora minima*, supported by strong in silico evidence. The compound exhibited superior binding affinities for key enzymes, α-amylase and α-glucosidase, compared to the standard drug acarbose. Despite minor violations of drug-likeness rules, stable interactions in molecular dynamics simulations and favorable ADMET properties further corroborate its development as an antidiabetic drug lead. Its structural similarity to native fucoxanthin, with a key modification at C8′, appears to enhance bioactivity while preserving biocompatibility. These findings support further in vitro and in vivo studies to validate efficacy, investigate pharmacokinetics, and explore its potential as a safe and effective therapeutic agent for the management of type 2 diabetes mellitus.

## Figures and Tables

**Table 1:** Total phenolic and flavonoid contents of crude methanol extracts and fractions of *Chnoospora minima*.

Extract/Fraction	TPC (mg GAE/g)	TFC (mg QE/g)
Crude methanol extract	57.01 ± 6.12 ^a^	0.79 ± 0.04 ^d^
Hexane fraction	2.96 ± 0.41 ^d^	0.21 ± 0.06 ^e^
Chloroform fraction	36.42 ± 2.74 ^b^	3.31 ± 0.04 ^b^
Ethyl acetate fraction	58.11 ± 4.28 ^a^	5.24 ± 1.01 ^a^
Aqueous fraction	19.90 ± 2.11 ^c^	1.05 ± 0.07 ^c^

TPC: Total phenol content; TFC: total flavonoid content; GAE: gallic acid equivalent; QE: quercetin equivalent. Data presented as mean standard deviation *(n* = 3). Mean values in a column superscripted by different letters (^a–e^) are significantly different at *p* < 0.05.

**Table 2:** IC_50_ values exhibited by C. *minima* methanol extract and methanol fractions for inhibition of α-amylase and α-glucosidase.

Extract/Fraction	α- Amylase (µg/mL)	α- Glucosidase (µg/mL)
Ethyl acetate fraction	30.56 ± 0.56 ^d^	14.78 ± 0.26 ^d^
Chloroform fraction	5.34 ± 0.32 ^e^	6.02 ± 0.18 ^e^
Aqueous fraction	92.12 ± 1.20 ^b^	36.92 ± 1.06 ^c^
Hexane fraction	149.31 ± 0.94 ^a^	83.92 ± 0.54 ^a^
Crude methanol extract	45.63 ± 0.04 ^d^	58.88 ± 2.01 ^b^
Acarbose	72.41 ± 0.24 ^c^	1.02 ± 0.07 ^f^

Data presented as mean standard deviation *(n* = 3). Mean values in a column superscripted by different letters (^a–f^) are significantly different at *p* < 0.05.

**Table 3:** ^1^H (600 MHz) and ^13^C (150 MHz) NMR spectra of the purified fucoxanthin derivative were recorded at room temperature in d-CDCl_3_, with J values reported in Hz.

Position	^13^C δ (ppm)	^1^H δ (ppm), Integration, Multiplicity, J (Hz)	Position′	13C δ (ppm)	^1^H δ (ppm), Integration, Multiplicity, J (Hz)
**C1**	35.14	-	**C1′**	35.76	-
**C2**	47.07	1.33 (^1^H, dd, J = 12.3) 1.47 (^1^H, dd, J = 13.8)	**C2′**	45.42	1.39 (^1^H, t, J = 12.63) 1.97 (^1^H, m)
**C3**	64.31	3.79 (^1^H, m)	**C3′**	67.97	5.36 (^1^H, tt J = 4.29, 11.38)
**C4**	41.66	1.76 (^1^H, dd, J = 9.18, 13.8) 2.30 (^1^H, ddd, J = 1.44, 4.74, 9.18)	**C4′**	45.22	1.49 (^1^H, t, J = 12.92) 2.26 (^1^H, ddd, J = 2.04, 4.2, 12.84)
**C5**	66.12	-	**C5′**	72.67	-
**C6**	67.06	-	**C6′**	117.51	-
**C7**	40.8	3.63, 2.58 (^2^H, d, J = 18.3)	**C7′**	103.36	6.03 (^1^H, s)
**C8**	197.83	-	**C8′**	202.33	-
**C9**	134.52	-	**C9′**	132.46	6.61 (1H, dd, J = 11.64, 14.16)
**C10**	139.06	7.13 (^1^H, d, J = 10.92)	**C10′**	128.51	6.11 (^1^H, d, J = 11.52)
**C11**	123.36	6.55 (^1^H, dd, J = 12.05, 15)	**C11′**	125.66	6.73 (^1^H, dd, J = 11.94, 14.6)
**C12**	144.99	6.65 (^1^H, d, J = 14.64)	**C12′**	137.09	6.25 (^1^H, d, J = 11.7)
**C13**	135.41	-	**C13′**	138.05	6.65 (^1^H, dd, J = 11.94, 14.64)
**C14**	136.6	6.39 (^1^H, dd, J = 11.58)	**C14′**	132.15	6.39 (^1^H, d, J = 11.58)
**C15**	129.4	6.61 (^1^H, dd, J = 11.64, 14.16)	**C15′**	132.13	6.55 (^1^H, dd, J = 12.05, 15)
**C16**	25.03	1.02 (^3^H, s)	**C16′**	32.07	1.05 (^3^H, s)
**C17**	28.11	0.94 (^3^H, s)	**C17′**	29.18	1.36 (^3^H, s)
**C18**	21.14	1.20 (^3^H, s)	**C18′**	31.27	1.33 (^3^H, s)
**C19**	11.8	1.92 (^3^H, s)	**C19′**	13.99	1.79 (^3^H, s)
**C20**	12.67	1.97 (^3^H, s)	**C20′**	12.95	1.97 (^3^H, s)
			**C21′**	170.38	2.02 (^3^H, s)
			**C22′**	21.4	-

**Table 4:** Predicted Pharmacokinetic and Drug-Likeness properties of Fucoxanthin derivative.

Class	Property	Value
Physicochemical Property	Molecular Weight	674.42
	nHA	7
	nHD	2
	nRot	13
	TPSA	113.43
	logS	−5.261
	logP	4.068
Drug likeness	Lipinski Rule	1 violation
	Vebers rule	1 violation
Absorption	Caco-2 Permeability	−4.947
	HIA	Yes
	Pgp-inhibitor	0.894
Distribution	PPB	78.242
	BBB	0
Metabolism	CYP1A2 inhibitor	Excellent
	CYP1A2 substrate	Excellent
	CYP2C19 inhibitor	Poor
	CYP2C19 substrate	Excellent
	CYP2C9 inhibitor	Poor
	CYP2C9 substrate	Excellent
	CYP2D6 inhibitor	Excellent
	CYP2D6 substrate	Excellent
	CYP3A4 inhibitor	Medium
	CYP3A4 substrate	Yes
Excretion	CLplasma	7.864
	T1/2	0.744
Toxicity	Toxicity Class	3
	AMES Mutagenicity	0.708
	Skin Sensitization	Yes
	Respiratory	No
	Eye Irritation	No

Number of hydrogen bond acceptors (nHA), number of hydrogen bond donors (nHD), number of rotatable bonds (nRot), topological polar surface area (TPSA), aqueous solubility (logS), octanol/water distribution coefficient (logP), human intestinal absorption (HIA), plasma protein binding (PPB), blood–brain barrier (BBB).

**Table 5:** Ramachandran plot statistics for optimized α-amylase and α-glucosidase models.

Protein Structure	Total No. of Residues	Favored Region	Allowed Region	Disallowed Region
α-amylase	460	449 (97.61%)	11 (2.39%)	0 (0%)
α-glucosidase	803	771 (96.02%)	30 (3.74%)	2 (0.25%)

**Table 6:** Molecular docking results of the fucoxanthin derivative and standard drug (Acarbose) with α-amylase and α-glucosidase.

Protein	Ligand	Binding Affinity (Kcal/mol)	No. of H-Bonds	H-Bonds Forming Residues (Bond Distance in A^0^)	No. of Non-Bonded Contacts
α-Amylase	Fucoxanthin Derivative	−9.4	3	SER^108^ (2.80), HSD^305^ (3.19), GLY^306^ (3.28)	59
	Acarbose	−8.5	5	GLU^282^ (2.80), ASP^402^ (2.89), ASP^402^ (3.09), GLY^403^ (3.13), ARG^421^ (3.08)	55
α-Glucosidase	Fucoxanthin Derivative	−8.0	1	VAL^718^ (2.84)	67
	Acarbose	−7.4	5	ASP^91^ (2.91), ALA^93^ (2.83), PRO^94^ (2.71), GLN^118^ (3.12), GLN^118^ (3.01)	61

## Data Availability

All data relevant to the publication are included.

## Abbreviations

The following abbreviations are used in this manuscript:

FD	Fucoxanthin Derivative
T2DM	Type 2 Diabetes Mellitus
RMSD	Root Mean Square Deviation
RMSF	Root Mean Square Fluctuation
SASA	Solvent Accessible Surface Area
TPSA	Topological Polar Surface Area
HIA	Human Intestinal Absorption
BBB	Blood–Brain Barrier
PPB	Plasma Protein Binding
HPLC	High Performance Liquid Chromatography
NMR	Nuclear Magnetic Resonance
H NMR	Hydrogen Nuclear Magnetic Resonance
^1^H-^1^H COSY	^1^H-^1^H Correlation Spectroscopy
HMQC	Hetero Nuclear Multiple Bond Correlation Spectroscopy
HMBC	^1^H-^13^C Heteronuclear Multiple Bond Correlation Spectroscopy
C NMR	Carbon Nuclear Magnetic Resonance

## References

[1] Oyewusi H.A., Wu Y.-S., Safi S.Z., Wahab R.A., Hatta M.H.M., Batumalaie K. (2023). Molecular dynamics simulations reveal the inhibitory mechanism of Withanolide A against α-glucosidase and α-amylase. J. Biomol. Struct. Dyn..

[2] Dahiru M.M., Musa N., Abaka A.M., Abubakar M.A. (2023). Potential Antidiabetic Compounds from *Anogeissus leiocarpus*: Molecular Docking, Molecular Dynamic Simulation, and ADMET Studies. Borneo J. Pharm..

[3] Goyal S., Rani J., Bhat M.A., Vanita V. (2023). Genetics of diabetes. World J. Diabetes.

[4] American Diabetes Association (2020). Obesity management for the treatment of type 2 diabetes: Standards of medical care in diabetes—2020. Diabetes Care.

[5] Rannan-Eliya R.P., Wijemunige N., Perera P., Kapuge Y., Gunawardana N., Sigera C., Jayatissa R., Herath H.M.M., Gamage A., Weerawardena N. (2023). Prevalence of diabetes and pre-diabetes in Sri Lanka: A new global hotspot-estimates from the Sri Lanka Health and Ageing Survey 2018/2019. BMJ Open Diabetes Res. Care.

[6] ElSayed N.A., Aleppo G., Bannuru R.R., Bruemmer D., Collins B.S., Ekhlaspour L., Gaglia J.L., Hilliard M.E., Johnson E.L., American Diabetes Association Professional Practice Committee (2024). 2. Diagnosis and Classification of Diabetes: Standards of Care in Diabetes—2024. Diabetes Care.

[7] Fatema K., Sharmin A.A., Sharna J.F., Haque A., Rahman M.M., Sarker S., Kazi M., Rahman R., Namakka M., Uzzaman M. (2024). Antioxidant and Antidiabetic Effects of *Flemingia macrophylla* Leaf Extract and Fractions: In vitro, Molecular Docking, Dynamic Simulation, Pharmacokinetics, and Biological Activity Studies. Bioresources.

[8] Kawee-Ai A., Kim A.T., Kim S.M. (2019). Inhibitory activities of microalgal fucoxanthin against α-amylase, α-glucosidase, and glucose oxidase in 3T3-L1 cells linked to type 2 diabetes. J. Oceanol. Limnol..

[9] Gunathilaka T., Keertihirathna L.R., Peiris D. (2022). Advanced Pharmacological Uses of Marine Algae as an Anti-Diabetic Therapy. Nat. Med. Plants.

[10] Jang H., Lee J., Park Y.-K., Lee J.-Y. (2024). Exploring the health benefits and concerns of brown seaweed consumption: A comprehensive review of bioactive compounds in brown seaweed and its potential therapeutic effects. J. Agric. Food Res..

[11] Gunathilaka T.L., Samarakoon K., Ranasinghe P., Peiris L.D.C. (2020). Antidiabetic Potential of Marine Brown Algae—A Mini Review. J. Diabetes Res..

[12] Gunathilaka T.L., Bandaranayake U., Boudjelal M., Ali R., Silva R.M., Samarakoon K.W., Ranasinghe P., Peiris L.D.C. (2024). Chnoospora minima: A Robust Candidate for Hyperglycemia Management, Unveiling Potent Inhibitory Compounds and Their Therapeutic Potential. Mar. Biotechnol..

[13] Mohibbullah M., Haque N., Sohag A.A.M., Hossain T., Zahan S., Uddin J., Hannan A., Moon I.S., Choi J.-S. (2022). A Systematic Review on Marine Algae-Derived Fucoxanthin: An Update of Pharmacological Insights. Mar. Drugs.

[14] Peng J., Yuan J.-P., Wu C.-F., Wang J.-H. (2011). Fucoxanthin, a marine carotenoid present in brown seaweeds and diatoms: Metabolism and bioactivities relevant to human health. Mar. Drugs.

[15] Abdulazeez S. (2019). Molecular simulation studies on B-cell lymphoma/leukaemia 11A (BCL11A). Am. J. Transl. Res..

[16] Jin Y., Arroo R. (2023). The protective effects of flavonoids and carotenoids against diabetic complications—A review of in vivo evidence. Front. Nutr..

[17] Aryal D., Joshi S., Thapa N.K., Chaudhary P., Basaula S., Joshi U., Bhandari D., Rogers H.M., Bhattarai S., Sharma K.R. (2024). Dietary phenolic compounds as promising therapeutic agents for diabetes and its complications: A comprehensive review. Food Sci. Nutr..

[18] Widyawati T., Yusoff N.A., Bello I., Asmawi M.Z., Ahmad M. (2022). Bioactivity-Guided Fractionation and Identification of Antidiabetic Compound of *Syzygium polyanthum* (Wight.)’s Leaf Extract in Streptozotocin-Induced Diabetic Rat Model. Molecules.

[19] Kumarasinghe H., Gunathilaka M. (2024). A systematic review of fucoxanthin as a promising bioactive compound in drug development. Phytochem. Lett..

[20] Ntie-Kang F., Mbah J.A., Lifongo L.L., Owono L.C.O., Megnassan E., Mbaze L.M., Judson P.N., Sippl W., Efange S.M. (2013). Assessing the pharmacokinetic profile of the CamMedNP natural products database: An in silico approach. Org. Med. Chem. Lett..

[21] Wang C., Kim J.H., Kim S.W. (2014). Synthetic biology and metabolic engineering for marine carotenoids: New opportunities and future prospects. Mar. Drugs.

[22] Shikov A.N., Flisyuk E.V., Obluchinskaya E.D., Pozharitskaya O.N. (2020). Pharmacokinetics of marine-derived drugs. Mar. Drugs.

[23] Fu L., Shi S., Yi J., Wang N., He Y., Wu Z., Peng J., Deng Y., Wang W., Wu C. (2024). ADMETlab 3.0: An updated comprehensive online ADMET prediction platform enhanced with broader coverage, improved performance, API functionality and decision support. Nucleic Acids Res..

[24] Sippel K.H., Quiocho F.A. (2015). Ion-dipole interactions and their functions in proteins. Protein Sci..

[25] Alafnan A., Chettupalli A.K., Unnisa A., Hussain T., Anwar S., Alkhojali W.M., Khalifa N.E., Osman M.E.D., Younes K.M., Abouzied A.S. (2023). In silico elucidation of plausible anti-obesity activity by Withaferin-A compound targeting alpha-amylase. Eur. Rev. Med. Pharmacol. Sci..

[26] Jung H.A., Ali M.Y., Choi R.J., Jeong H.O., Chung H.Y., Choi J.S. (2016). Kinetics and molecular docking studies of fucosterol and fucoxanthin, BACE1 inhibitors from brown algae *Undaria pinnatifida* and *Ecklonia stolonifera*. Food Chem. Toxicol..

[27] Khenifi M.L., Serseg T., Migas P., Krauze-Baranowska M., Özdemir S., Bensouici C., Alghonaim M.I., Al-Khafaji K., Alsalamah S.A., Boudjeniba M. (2023). HPLC-DAD-MS Characterization, Antioxidant Activity, α-amylase Inhibition, Molecular Docking, and ADMET of Flavonoids from Fenugreek Seeds. Molecules.

[28] Zhang Y., Zhao W., Xing Z., Zhu B., Hou R., Zhang J., Li T., Zhang Z., Wang H., Li Z. (2022). Study on the binding behavior and functional properties of soybean protein isolate and β-carotene. Front. Nutr..

[29] Fusani L., Palmer D.S., Somers D.O., Wall I.D. (2020). Exploring Ligand Stability in Protein Crystal Structures Using Binding Pose Metadynamics. J. Chem. Inf. Model..

[30] Idris M.O., Yekeen A.A., Alakanse O.S., Durojaye O.A. (2020). Computer-aided screening for potential TMPRSS2 inhibitors: A combination of pharmacophore modeling, molecular docking and molecular dynamics simulation approaches. J. Biomol. Struct. Dyn..

[31] Khan A., Gui J., Ahmad W., Haq I., Shahid M., Khan A.A., Shah A., Khan A., Ali L., Anwar Z. (2021). The SARS-CoV-2 B.1.618 variant slightly alters the spike RBD-ACE2 binding affinity and is an antibody escaping variant: A computational structural perspective. RSC Adv..

[32] Salehi B., Sharifi-Rad J., Seca A.M.L., Pinto D.C.G.A., Michalak I., Trincone A., Mishra A.P., Nigam M., Zam W., Martins N. (2019). Current trends on seaweeds: Looking at chemical composition, phytopharmacology, and cosmetic applications. Molecules.

[33] Jeyaseelan E.C., Jeyaseelan T.C., Thavaranjit A.C. (2012). Antibacterial Activity of Some Selected Algae Present in the Costal Lines of Jaffna Peninsula. Int. J. Pharm. Biol. Arch..

[34] Lakmal H.C., Samarakoon K.W., Lee W., Lee J.-H., Abeytunga D., Lee H.-S., Jeon Y.-J. (2014). Anticancer and antioxidant effects of selected Sri Lankan marine algae. J. Natl. Sci. Found. Sri Lanka.

[35] Singleton V.L., Orthofer R., Lamuela-Ravent R.M. (1999). Analysis of Total Phenols and Other Oxidation Substrates and Antioxidants by Means of Folin-Ciocalteu Reagent. Methods Enzymol..

[36] Kokilam G., Vasuki S., Sajitha N. (2013). Biochemical composition, alginic acid yield and antioxidant activity of brown seaweeds from mandapam region, gulf of mannar. J. Appl. Pharm. Sci..

[37] Gunathilaka T.L., Samarakoon K.W., Ranasinghe P., Peiris L.C.D. (2019). In-Vitro Antioxidant, Hypoglycemic Activity, and Identification of Bioactive Compounds in Phenol-Rich Extract from the Marine Red Algae *Gracilaria edulis* (Gmelin) Silva. Molecules.

[38] Zhou B., Huang N., Zeng W., Zhang H., Chen G., Liang Z. (2020). Development of a strategy for the screening of α-glucosidase-producing microorganisms. J. Microbiol..

[39] Jawarkar R.D., Sharma P., Jain N., Gandhi A., Mukerjee N., Al-Mutairi A.A., Zaki M.E.A., Al-Hussain S.A., Samad A., Masand V.H. (2022). QSAR, Molecular Docking, MD Simulation and MMGBSA Calculations Approaches to Recognize Concealed Pharmacophoric Features Requisite for the Optimization of ALK Tyrosine Kinase Inhibitors as Anticancer Leads. Molecules.

[40] Yuan S., Chan H.C.S., Hu Z. (2017). Using PyMOL as a platform for computational drug design. Wiley Interdiscip. Rev. Comput. Mol. Sci..

[41] Kim S., Lee J., Jo S., Brooks C.L., Lee H.S., Im W. (2017). CHARMM-GUI ligand reader and modeler for CHARMM force field generation of small molecules. J. Comput. Chem..

[42] Kim S., Chen J., Cheng T., Gindulyte A., He J., He S., Li Q., Shoemaker B.A., Thiessen P.A., Yu B. (2023). PubChem 2023 update. Nucleic Acids Res..

[43] Xiong G., Wu Z., Yi J., Fu L., Yang Z., Hsieh C., Yin M., Zeng X., Wu C., Lu A. (2021). ADMETlab 2.0: An integrated online platform for accurate and comprehensive predictions of ADMET properties. Nucleic Acids Res..

[44] Banerjee P., Kemmler E., Dunkel M., Preissner R. (2024). ProTox 3.0: A webserver for the prediction of toxicity of chemicals. Nucleic Acids Res..

[45] Berman H.M., Westbrook J., Feng Z., Gilliland G., Bhat T.N., Weissig H., Shindyalov I.N., Bourne P.E. (2000). The Protein Data Bank. Nucleic Acids Res..

[46] Jo S., Kim T., Iyer V.G., Im W. (2008). CHARMM-GUI: A web-based graphical user interface for CHARMM. J. Comput. Chem..

[47] Dallakyan S., Olson A.J. (2015). Small-molecule library screening by docking with PyRx. Methods Mol. Biol..

[48] Trott O., Olson A.J. (2010). AutoDock Vina: Improving the speed and accuracy of docking with a new scoring function, efficient optimization, and multithreading. J. Comput. Chem..

[49] Laskowski R.A. (2009). PDBsum new things. Nucleic Acids Res..

[50] Phillips J.C., Hardy D.J., Maia J.D.C., Stone J.E., Ribeiro J.V., Bernardi R.C., Buch R., Fiorin G., Hénin J., Jiang W. (2020). Scalable molecular dynamics on CPU and GPU architectures with NAMD. J. Chem. Phys..

[51] Best R.B., Zhu X., Shim J., Lopes P.E.M., Mittal J., Feig M., MacKerell A.D. (2012). Optimization of the additive CHARMM all-atom protein force field targeting improved sampling of the backbone φ, ψ and side-chain χ_1_ and χ_2_ Dihedral Angles. J. Chem. Theory Comput..

[52] Jorgensen W.L., Chandrasekhar J., Madura J.D., Impey R.W., Klein M.L. (1983). Comparison of simple potential functions for simulating liquid water. J. Chem. Phys..

[53] Guterres H., Im W. (2020). Improving Protein-Ligand Docking Results with High-Throughput Molecular Dynamics Simulations. J. Chem. Inf. Model..

[54] Klauda J.B., Venable R.M., Freites J.A., O’Connor J.W., Tobias D.J., Mondragon-Ramirez C., Vorobyov I., MacKerell A.D., Pastor R.W. (2010). Update of the CHARMM All-Atom Additive Force Field for Lipids: Validation on Six Lipid Types. J. Phys. Chem. B.

